# Genetic variability in the *E6* and *E7* oncogenes of HPV52 and its prevalence in the Taizhou area, China

**DOI:** 10.1186/s12985-022-01929-5

**Published:** 2022-11-22

**Authors:** Zhi Yang, Zhe-Hang He, Yang Zhang, Xing-Hong Di, Die-Fei Zheng, Hui-Hui Xu

**Affiliations:** 1 Medical Research Center, Taizhou Hospital of Zhejiang Province, Wenzhou Medical University, Linhai, 317000 Zhejiang China; 2 Scientific Research Department, Taizhou Hospital of Zhejiang Province, Wenzhou Medical University, Linhai, 317000 Zhejiang China

**Keywords:** Human papillomavirus 52, Genetic variability, Phylogenetic analysis, Cervical carcinogenesis

## Abstract

**Background:**

Human papillomavirus (HPV) 52 is one of the prevalent oncogenic HPV genotypes in East Asia. Chinese women have the highest susceptibility to the HPV52 type, but research data on HPV52 genetic variability and its carcinogenicity in China is lacking.

**Methods:**

The present study aimed to investigate the genetic variability of HPV52 currently circulating among Chinese women by PCR sequencing the entire *E6* and *E7* oncogenes. HPV52 sequence alignment, genetic heterogeneity analyses and maximum-likelihood phylogenetic tree construction were performed by BioEdit software and MEGA X software.

**Results:**

Between 2016 and 2018, the overall HPV infection rate was 21.3%, of which HPV52 was the most prevalent high-risk type (17.2%) in the Taizhou area, China. A total of 339 single HPV52-positive samples were included in this study. We obtained 27 distinct variation patterns of HPV52 with the accession GenBank numbers ON529577-ON529603. Phylogenetic analysis showed that 96.6% of HPV52 variants belonged to lineage B, which seemed to be uniquely defined by G350T, A379G (K93R) in the *E6* gene and C751T, A801G in the *E7* gene. Due to the dominance of lineage B in our study population, the results could not be used to assess the association of the HPV52 (sub)lineage with the risk of cervical lesions. In addition, no significant trends were observed between the nucleotide substitutions of HPV52 variants and the risk of cervical carcinogenesis.

**Conclusion:**

Our data showed that HPV52 variants were strongly biased towards lineage B. These results confirmed that cervical lesions in the Taizhou area are highly attributable to HPV52, which may be due to the high infection rate of lineage B in the population.

**Supplementary Information:**

The online version contains supplementary material available at 10.1186/s12985-022-01929-5.

## Background

Cervical cancer caused by persistent infection with high-risk human papillomavirus (HPV) continues to be a global public health problem. According to GLOBOCAN 2018, there are approximately 106,400 new cases and 47,700 deaths related to cervical cancer annually in China, accounting for 18.6% and 15.2% of the global data, respectively [1]. To date, over 170 HPV types have been identified and mainly divided into high- and low-risk types based on the malignant progression of the disease [2]. Of the known high-risk HPV types, HPV16 is the most common type worldwide [3], but the infection rate of HPV52 in East Asia (such as China and Japan) is much higher than that in other areas around the world [4]. In our previous Taizhou area HPV studies, the results suggested that Chinese women have the highest susceptibility to the HPV52 type, but the carcinogenic ability of HPV52 was relatively weaker than that of HPV16 or HPV58 [5–7]. The genomic characterization of HPV52 has obvious epidemiological characteristics, especially the (sub)lineages and single nucleotide substitutions in the two major oncogenes *E6* and *E7*, which may increase the risk of infection with HPV52 in Chinese women [8–10].

HPV52 genomes are approximately 7.9 kb, and their gene structure includes the early regions (*E1, E2, E4, E5, E6,* and *E7*), the late regions (*L1* and *L2*) and the long control region (*LCR*). When HPV infects cervical epithelial cells and integrates into the host genome, the expression of two major *E6* and *E7* oncoproteins increases uncontrolled, resulting in host cell immortalization, transformation, and carcinogenesis [11]. Based on genomic DNA analysis, HPV52 variants are divided into five main phylogenetic lineages (A, B, C, D, and E) and nine sublineages (A1, A2, B1, B2, B3, C1, C2, D, and E) [12, 13]. The distribution of HPV variants varies with their geographical location and ethnic group, especially HPV52, which is more likely to infect Chinese women. However, there are few research data on HPV52 genetic variability and its carcinogenicity in China. In this study, we investigated the genetic variability in the *E6* and *E7* genes of HPV52 obtained from exfoliated cervical cells and explored their role in cervical cancer risk among Taizhou women in China.

## Methods

### Subject recruitment

From April 2016 to December 2018, exfoliated cervical cells were collected from outpatients undergoing cervical cancer screening at the gynaecological clinic of Taizhou Hospital, Zhejiang Province. The inclusion criteria for this study were as follows: single HPV52 infection. The exclusion criteria were as follows: second round of screening or more, received cervical treatment, and had a history of cancer or hysterectomy. The samples in cell preservation medium were stored at − 20 °C before DNA extraction.

According to several years of experience with cervical cancer screening in the Taizhou area, we recommended that HPV52-positive patients undergo colposcopy examination immediately [5]. The suspected cervical tissues were fixed in a 10% formalin solution, and then histological diagnoses were performed by two pathologists. According to the histological criteria of the World Health Organization (WHO), they were divided into cervical intraepithelial neoplasia (CIN) grade 1, CIN2, CIN3, cervical cancer or normal.

### DNA extraction and HPV genotyping

Total cellular DNA was extracted using a DNA Extraction Kit (#GK0122, GENEray, China). HPV genotyping was performed using the GP5 + /bioGP6 + -PCR/MPG assay (CFDA Certified No. (2017): 3,404,697). This assay can simultaneously classify 27 different HPV types, including 14 high-risk HPV types (HPV16, 18, 31, 33, 35, 39, 45, 51, 52, 56, 58, 59, 66, and 68) and 13 low-risk HPV types (HPV6, 11, 26, 40, 42, 43, 44, 53, 55, 61, 81, 82, and 83). The detailed operation process of HPV genotyping has been described in our previous studies [5, 6]. To avoid the influence of other HPV types on HPV52, only single HPV52-positive samples were selected for subsequent DNA amplification, sequencing and phylogenetic analysis in this study.

### DNA amplification and sequencing

The primer sequences specific for the *E6* and *E7* genes of HPV52 were 52E6E7_F 5′-ACCCACAACCACTTTTTTTTAT-3’ and 52E6E7_R 5′-TTGCCTCTACTTCAAACCAGCC-3’, which were cited from a recently published article [8]. Primers were synthesized by BGI company (Hangzhou, China). The 50 µl PCR volumes included 25 µl 2 × PrimeSTAR Max Premix (TaKaRa, Japan), 20 µl nuclease-free water, 2 µl forward primer, 2 µl reverse primer, and 1 µl template DNA. The PCR conditions were as follows: initial denaturation step at 95 °C for 5 min, followed by 35 repeated cycles of 94 °C for 30 s, 60 °C for 30 s, 72 °C for 30 s, a final extension step at 72 °C for 10 min, and holding at 4 °C.

The size of the PCR amplification product was 957 bp (nucleotide sites [nt] 7916–930, including the *E6* gene nt 102–548 and *E7* gene nt 553–852). Subsequently, the PCR amplification products were purified and sequenced on the ABI 3730xl DNA Genetic Analyser at BGI company. To avoid PCR or sequencing errors, all data were confirmed by repeating the PCR and sequencing reactions at least twice.

### Phylogenetic analysis

In this study, the HPV52 reference sequence (GenBank: HQ537732) was used as the standard for alignment and nucleotide site numbering. The variations in the *E6* and *E7* genes in the HPV52 sequences were aligned by BioEdit software. The HPV52 phylogenetic tree was constructed using the maximum-likelihood statistical method by MEGA X software [14]. One thousand bootstrap replicates were used for tree topology evaluation. To construct the phylogenetic branches, HPV52 sequences were downloaded from NCBI (https://www.ncbi.nlm.nih.gov/), including HQ537732 (A1), HQ537739 (A2), HQ537740 (B1), HQ537743 (B2), MZ374448 (B3), HQ537744 (C1), HQ537746 (C2), HQ537748 (D), and MZ374428 (E) [12, 13].

### Statistical analysis

The association of cervical lesion risk with HPV52 variants was analysed using the chi-square test or Fisher’s exact test. Odds ratios (ORs) and relative 95% confidence intervals (95% CI) were calculated. All statistical analyses were performed using SPSS version 16.0 (SPSS Inc., Chicago, IL). *P* values were two-sided, and the results were regarded as statistically significant if the *P* value was 0.05 or less.

## Results

### Characteristics of the study population

**Fig. 1:**
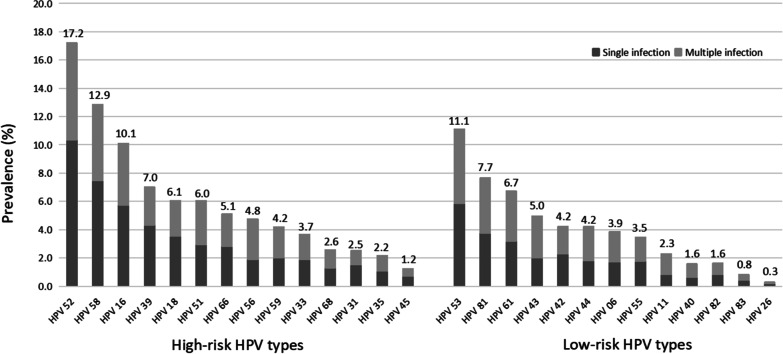
Prevalence of the HPV types in single and multiple infections in Taizhou area, Southeast China

Between April 2016 and December 2018, the overall HPV infection rate was 21.3% (3287/15429) in women who were screened for cervical cancer in Taizhou Hospital. Among all HPV-positive women, the infection rate of HPV subtypes is shown in Fig. 1. HPV52 was the most prevalent high-risk type (17.2%, 565/3287), followed by HPV58 (12.9%), HPV16 (10.1%), HPV39 (7.0%), HPV18 (6.1%), and HPV51 (6.0%). The top six low-risk HPV types were HPV53 (11.1%), HPV81 (7.7%), HPV61 (6.7%), HPV43 (5.0%), HPV42 (4.2%), and HPV44 (4.2%).

In the present study, 339 women (median age 40.8 years; range 19–75) with a single HPV52 infection were selected (Additional file 1). A total of 325 (95.9%) sequences of the entire *E6* and *E7* genes from HPV52 were obtained. Fourteen (4.1%) sequences were excluded due to the small number of HPV copies. Among 325 HPV52-infected women, 75 (23.1%) had normal and adequate cervices by colposcopy examination; therefore, no further biopsy diagnosis was performed. Sixty-one (18.8%) women refused further colposcopy examination. The remaining 189 (58.1%) underwent colposcopy biopsy for diagnosis, which was further used for risk association analysis, and 67 were diagnosed with normal cervices after biopsy, 53 with CIN1, 45 with CIN2, 24 with CIN3, and no cases of cervical cancer. The characteristics of the study population categorized by HPV52 (sub)lineage are shown in Table 1.

**Table 1:** Characteristics of the study population categorized by HPV52 (sub)lineage

HPV52 (sub)lineages	Normal (n = 67)	CIN1 (n = 53)	CIN2 (n = 45)	CIN3 (n = 24)	Cancer (n = 0)	Total (n = 189)
A1	2 (3.0%)	1 (1.9%)	1 (2.2%)	0	0	4 (2.1%)
A2	0	0	0	0	0	0
B	64 (95.5%)	52 (98.1%)	42 (93.3%)	23 (95.8%)	0	181 (95.8%)
52CNTZ05	39 (58.2%)	31 (58.5%)	26 (57.8%)	12 (50.0%)	0	108 (57.1%)
52CNTZ12	16 (23.9%)	15 (28.3%)	8 (17.8%)	8 (33.3%)	0	47 (24.9%)
52CNTZ17	2 (3.0%)	2 (3.8%)	2 (4.4%)	0	0	6 (3.2%)
52CNTZ21	2 (3.0%)	0	3 (6.7%)	0	0	5 (2.6%)
Other	5 (7.5%)	4 (7.5%)	3 (6.7%)	3 (12.5%)	0	15 (7.9%)
C1	0	0	0	0	0	0
C2	1 (1.5%)	0	2 (4.4%)	1 (4.2%)	0	4 (2.1%)
D	0	0	0	0	0	0

### Variations in the *E6* and *E7* genes

**Fig. 2:**
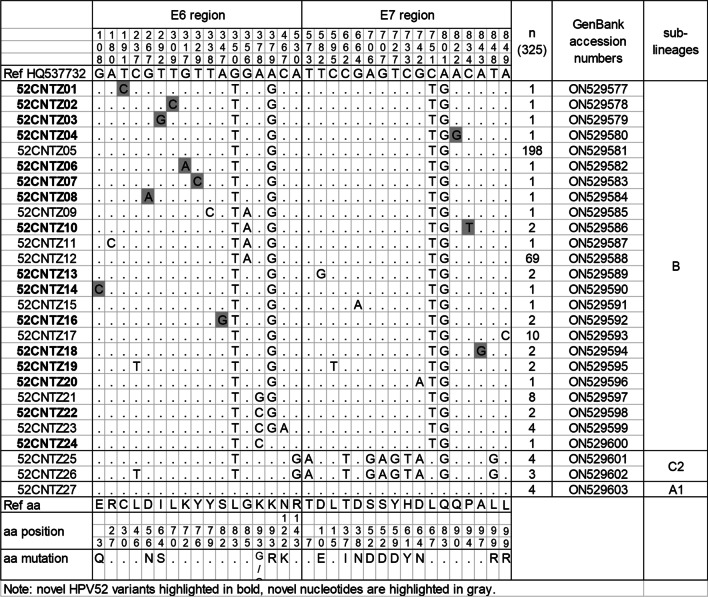
Genetic variability of HPV52 *E6* and *E7* nucleotide sequences in Taizhou area, Southeast China. Numbering refers to the first nucleotide of the HPV52 prototype reference sequence (GenBank: HQ537732). Each row indicates the variant identification and the nucleotide sequence alignment compared to the reference. Novel HPV52 variants are highlighted in bold and novel nucleotide substitutions are highlights in gray

Compared with the HPV52 reference sequence HQ537732, 98.8% (321/325) of collected HPV52 samples showed nucleotide variations. To visually distinguish the specific nucleotide variations of HPV52 lineage/sublineages, we summarized all of the changes in nucleotide and amino acid sequences in the entire *E6* and *E7* fragments, as shown in Fig. 2. In this study, we obtained 27 distinct variation patterns denoted 52CNTZ01-52CNTZ27, which were published with the GenBank accession codes ON529577 to ON529603. Of these, 16 (59.3%, 16/27) novel HPV52 variants were detected and are highlighted in bold in Fig. 2. 52CNTZ05 (60.9%, 198/325) was the most common variant in the Taizhou-based population, followed by 52CNTZ12 (21.2%, 69/325) and 52CNTZ17 (3.1%, 10/325).

In the *E6* and *E7* genes, a total of 35 single nucleotide substitutions were identified, with 17 (48.6%) nonsynonymous substitutions and 11 (31.4%) novel substitutions. The four most prevalent nucleotide substitutions were G350T (321/325, 98.8%), A379G (K93R) (313/325, 96.3%) in the *E6* gene and C751T (314/325, 96.6%), A801G (321/325, 98.8%) in the *E7* gene. These four nucleotide substitutions were specific to HPV52 lineage B, but no significant trends in the severity of cervical lesions were observed. Notably, there were three amino acid changes in the 93rd genetic code of the E6 oncoprotein: K93G, K93Q, and K93R. The corresponding nucleotide changes were A378G, A378C, and A379G, which belong to the B lineage in this study. To the best of our knowledge, the nucleotide substitutions of G108C (E3Q), T191C, G267A (D56N), T292G (I64S), T309C, G317A, T329C, and A347G in the *E6* gene and A822G, C834T, and A843G in the *E7* gene have not been reported in previous studies. However, these novel nucleotide substitutions were detected in only one or two samples, and thus, no statistical analysis was performed.

### Phylogenetic construction

**Fig. 3:**
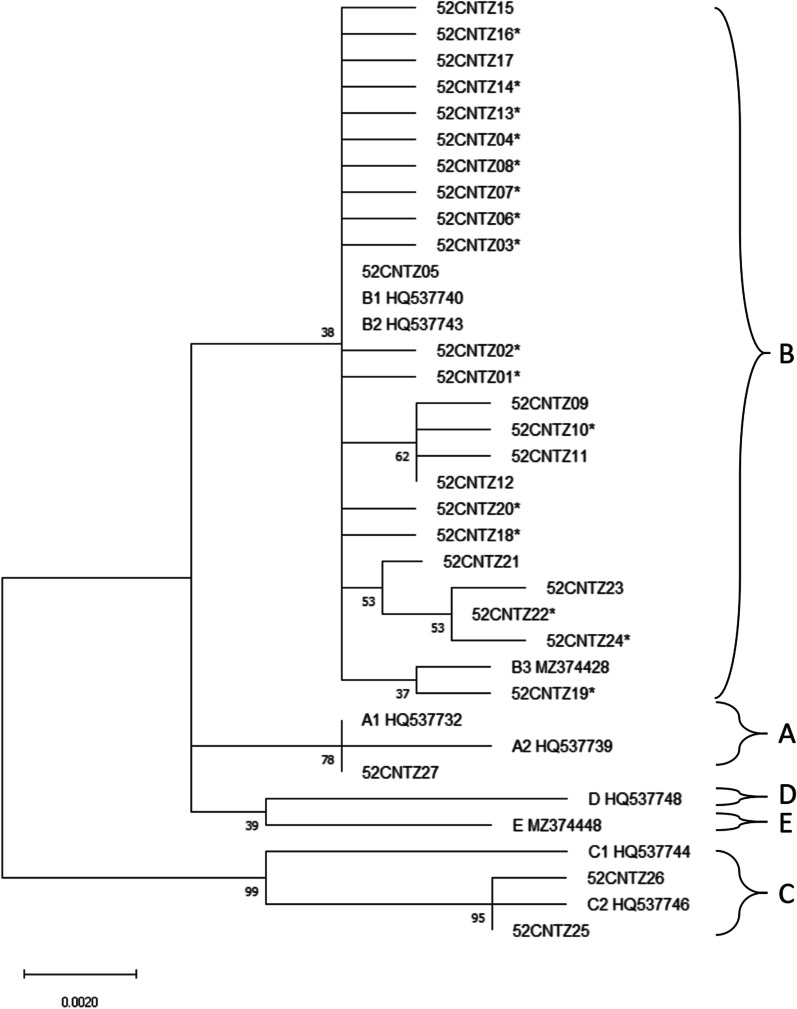
Phylogenetic tree of the HPV52 variants. Maximum-likelihood analysis (with MEGA X) of *E6* and *E7* nucleotide sequences was inferred from 27 obtained HPV52 variants and 9 reference sequences. Numbers below branches indicate bootstrap values

The maximum-likelihood phylogenetic tree based on the HPV52 *E6-E7* sequences was inferred from 27 obtained HPV52 variants and 9 reference sequences (Fig. 3). According to the phylogenetic tree, the most predominant HPV52 variants belong to lineage B (96.6%, 314/325), followed by sublineages C2 (2.2%, 7/325) and A1 (1.2%, 4/325). Lineage B appeared to be uniquely defined by G350T and A379G (K93R) in the *E6* gene and C751T and A801G in the *E7* gene. 52CNTZ27 in the A1 sublineage, 52CNTZ19 in the B3 sublineage, and 52CNTZ25 and 52CNTZ26 in the C2 sublineage had proportions of 1.2%, 0.6%, and 2.2%, respectively.

### Risk association with cervical lesions

In this study, 189 HPV52-infected women underwent colposcopy biopsy for diagnosis, including 67 normal cervices, 53 with CIN1, 45 with CIN2, and 24 with CIN3. These cases were further used to analyse the association between sequence variations of HPV52 and the risk of cervical lesions. Given the dominance of HPV52 lineage B in our population, the results showed that for HPV52, any association between a specific (sub)lineage and a higher risk of CIN2 or worse lesions could not be well evaluated. Other (sub)lineages were not included in these analyses due to small numbers of samples. Furthermore, no significant difference in the relative risk for cervical carcinogenesis progression was observed among the nucleotide variations in HPV52 *E6* and *E7* oncogenes (Table 1).

## Discussion

Persistent high-risk HPV infection has been identified as a major risk factor for cervical cancer. In China, the infection rates of high-risk HPV, such as HPV16, 18, 52, and 58, increase with the severity of cervical lesions [5, 7]. The overall HPV infection rate (21.3%) of this study was significantly different from that of Yangqu (8.9%), Xinjiang (14.0%), and Shandong (28.4%) but similar to that of other regions in Southeast China, such as Hangzhou (22.4%) and Jiangxi (22.5%) [15–19]. Many factors, including different economic conditions, cultural diversity, HPV vaccine awareness and lifestyles, might be the reasons for the differences in HPV infection rates in different geographical regions [7, 20, 21].

In this study, HPV52 was the most prevalent HPV type, followed by HPV58, 53, 16, 81, 39, 61, and 18. It is worth noting that the Gardasil 9-valent prophylactic vaccine approved for marketing in China in 2016 mainly targets HPV 6, 11, 16, 18, 31, 33, 45, 52, and 58. Therefore, the development of polygenic vaccines containing HPV53, 81, 39, and 61 would be more helpful for Chinese women to prevent HPV infection. Among all HPV types in China, the infection rate of HPV52 was relatively low in the late 1990s but has gradually increased since then [6, 7, 22]. Recently, HPV52 has become the most prevalent type among the Chinese population [6, 8, 9, 17–19, 30]. Furthermore, the oncogenicity of HPV is mainly attributable to the combined effects of the E6 and E7 oncoproteins. Accordingly, therapeutic vaccines against HPV E6 and/or E7 have been widely studied [23]. Based on our previous findings, genetic variation in the *E6* and *E7* genes was found to be highly associated with the risk of cervical cancer [24–26]. The genomic characterization of the HPV52 type has obvious epidemiological characteristics, which may be one of the factors that increase the risk of cervical cancer in Chinese women.

We obtained 325 complete sequences of the *E6* and *E7* genes from HPV52 in the Taizhou area. Most of the HPV52 (96.6%) variants belonged to lineage B, which was consistent with previous reports on HPV52 (sub)lineage distribution in East Asia [27–30]. It has been reported that lineage B predominated in Asia, while lineage A was the most common lineage in Africa, the Americas and Europe [27]. Given the dominance of HPV52 lineage B detected in our population, the results showed that for HPV52, any association between a specific (sub)lineage and a higher risk of CIN2 or worse lesions could not be well evaluated. Other (sub)lineages were not included in these analyses due to small numbers of samples. In addition to the HPV52 variants of the B lineage, the remaining 52CNTZ27 belongs to the A1 sublineage, and 52CNTZ25 and 52CNTZ26 belong to the C2 sublineage, accounting for 1.2% and 2.2%, respectively. A previous study showed that lineage B had a higher risk of cervical cancer than lineage A (OR: 5.46, 95% CI 2.28–13.07) [27]. Therefore, our results suggested that the reported high attribution of disease to HPV52 in the Taizhou area may be due to the high infection rate of lineage B in our population. However, our existing results could not be used to assess the association of the HPV52 (sub)lineage with the risk of cervical lesions because HPV52 variants were strongly biased towards lineage B. A study investigating the worldwide distribution of HPV52 variants suggested that all lineage B variants identified were sublineage B2, with 89.0% in Asia, 5.5% in Americas, 1.1% in Europe and zero in Afric [27]. Studies from Japan and Korea on HPV52 variants also suggested that all lineage B variants identified were sublineage B2 [29, 31]. During the long history of virus evolution, these HPV52 variants of lineage B may match the characteristics of the East Asia population through unknown viral adaptation mechanisms.

Compared with the HPV52 reference sequence (GenBank: HQ537732), the four most prevalent nucleotide substitutions were G350T (98.8%), A379G (K93R) (96.3%) in the *E6* gene and C751T (96.6%), A801G (98.8%) in the *E7* gene, which were specific to HPV52 lineage B. Of these, K93R was the only nonsynonymous substitution. Notably, in this study, there were three amino acid changes in the 93rd genetic code of the E6 oncoprotein, K93G, K93Q, and K93R, but no significant trends between the nucleotide substitutions of HPV52 variants and the risk for cervical carcinogenesis were observed. Previous studies have shown that K93R was associated with an increased cervical lesion risk in Korean women, but it has no correlation in Chinese women [31–33]. The sequence variants of amino acids may lead to different levels of pathogenicity due to geographical location or ethnic discrepancies. An in vitro study found that although K93R did not increase the immortalization ability of HPV52-positive cells, stronger colony formation and greater cell migration ability were observed when compared to its prototype and other variants [27]. In addition, the roles of synonymous substitutions G350T, C751T and A801G in lineage B on the risk of cervical cancer need to be further studied. Moreover, further functional studies should be conducted to understand the molecular mechanism of how these HPV52 variants of lineage B contribute to enhanced carcinogenicity, together with elucidation of the genetic background of Chinese people, including human leukocyte antigen polymorphisms.

## Conclusions

In summary, this is the first comprehensive study to evaluate the genetic variants and phylogenetics of the HPV52 *E6* and *E7* genes in the Taizhou area, Southeast China. Our study confirmed that the common HPV52 infection in the Taizhou area of China when compared to other areas around the world is largely due to the high infection rate of lineage B in the population.

## Supplementary Information

**Additional file 1**. Clinical data for HPV52 study in Taizhou area, China.

## Data Availability

All data generated during this study are included in this published article. The supplementary materials included the nucleotide variations of the *E6* and *E7* genes from HPV52 and the follow-up data of patients. In addtion, these sequences have been released to GenBank database with the accession codes of ON529577-ON529603. The links are https://www.ncbi.nlm.nih.gov/nuccore/ON529577 ~ https://www.ncbi.nlm.nih.gov/nuccore/ON529603.

## Abbreviations

bp: Base pair
CIN: Cervical intraepithelial neoplasia
DNA: Deoxyribonucleic acid
HPV: Human papillomavirus
LCR: Long control region
LEEP: Loop electrosurgical excision procedure
nt: Nucleotide sites
OR: Odds ratios
ORF: Open reading frames
PCR: Polymerase chain reaction
SNP: Single nucleotide polymorphism
WHO: World Health Organization
